# Radial Arterial Cannulation by Ultrasound-Guided Dynamic Needle-Tip Positioning Using the Short-Axis Out-of-Plane Approach Versus the Long-Axis In-Plane Approach: A Randomized Controlled Study

**DOI:** 10.7759/cureus.54183

**Published:** 2024-02-14

**Authors:** Bharath Kumar Mesa, Mamta Sinha, Mayank Kumar, Sarita Ramchandani, Chandan Dey, Nandkishore Agrawal, Monica Khetarpal

**Affiliations:** 1 Anesthesiology, All India Institute of Medical Sciences, Raipur, Raipur, IND; 2 Emergency Medicine and Trauma, All India Institute of Medical Sciences, Raipur, Raipur, IND

**Keywords:** first pass success, long axis approach, short axis approach, dynamic needle tip positioning, ultrasound, radial artery cannulation

## Abstract

Introduction

Radial artery cannulation is a commonly performed invasive procedure for assessing a patient's hemodynamic status and collecting blood samples. Ultrasound guidance has shown benefits in improving the success rate of first-attempt cannulation. Two main approaches, short-axis out-of-plane (SAOOP) and long-axis in-plane (LAIP), are commonly used. A modified technique called dynamic needle-tip positioning (DNTP) using the short-axis out-of-plane approach has been reported to enhance arterial catheterization. This study aims to compare the first-attempt success rates of radial artery cannulation using the two techniques, DNTP versus LAIP, along with overall success rates, cannulation time, and number of attempts.

Methods

This prospective, randomized, controlled, clinical study was conducted after obtaining clearance from the Institute Ethics Committee of AIIMS, Raipur. Ninety-six patients between the ages of 18 and 50 years, undergoing elective surgery under general anesthesia, and required radial arterial cannulation were randomized and equally allocated into two groups as the LAIP and DNTP approaches. The first-pass success rate, time to achieve successful cannulation, number of attempts needed, overall success rate within five minutes, and potential complications, such as thrombosis, vasospasm, and hematoma, were recorded.

Results

A total of 96 patients were included, with 48 in the LAIP group and 48 in the DNTP group. The DNTP group showed statistically significant advantages over the LAIP group, with a higher first-pass success rate (97.9% vs. 83.3%; p = 0.014) and shorter time to achieve successful cannulation (9.29±3.79 vs. 26.16±20.22 seconds; p = 0.001).

Conclusion

The ultrasound-guided short-axis DNTP technique for radial artery cannulation demonstrated a significant advantage as compared to the LAIP technique. The DNTP technique resulted in higher first-attempt cannulation success and shorter cannulation time.

## Introduction

Percutaneous arterial catheterization is a commonly done invasive procedure in the operating room to evaluate the hemodynamic status of the patient and to collect blood samples frequently. The radial artery is the preferred choice for arterial cannulation due to its ease of access, high success rate, and the maintenance of dual blood supply to the hands through the ulnar artery [1].

Though major problems are uncommon, cannulation of the radial artery can be difficult, especially in hypotensive and peripheral vascular disease patients [2]. Several failed arterial cannulation attempts increase patient discomfort and increase the risk of adverse outcomes such as arterial spasm, thrombosis, and local hematoma.

Recently, ultrasound-guided arterial cannulations have demonstrated an improved rate of first-attempt cannulation success and less incidence of cannulation-related complications [3,4]. It has also been found useful in emergency scenarios like cannulation in a prone position [5].

The short-axis out-of-plane (SAOOP) approach and the long-axis in-plane (LAIP) approach are the two major approaches used in ultrasound sonography (USG)-guided vascular catheterization [6,7]. In recent times, a modified ultrasound approach where the needle tip is progressed in a stepwise fashion, i.e., DNTP using the short-axis out-of-plane approach has been described, which is shown to result in better arterial catheterization. The DNTP approach enables precise imaging of the needle tip by alternatively adjusting the needle and ultrasound probe using the short-axis out-of-plane technique. It has thus been described as useful for the catheterization of peripheral vascular structures in both adults and the pediatric population [8,9].

As limited studies have been done to compare DNTP using the short-axis out-of-plane approach and the long-axis in-plane approach for radial artery cannulation, the study was planned.

The primary aim of this study was to compare the first-attempt success rate of radial artery cannulation between DNTP using the short-axis out-of-plane approach and the conventional LAIP approach. The secondary aim was to compare the overall success rate within five minutes, the cannulation time, the number of attempts required, and the complications that occurred between the two techniques.

## Materials and methods

Following approval from the institute ethical committee (1050/IEC-AIIMSRPR/2020) and registration with the Clinical Trials Registry of India (CTRI/2021/05/033757), the study was initiated. The study design was a prospective, randomized controlled clinical trial. Participants undergoing elective surgery who required invasive blood pressure monitoring in the age group of 18-50 years and of American Society of Anesthesiologists (ASA) physical status I, II, or III were included in the study after taking written, informed consent. Patients with a history of forearm surgery, radial artery cannulation in the previous 30 days, negative Allen test, obesity (BMI >30 kg/m^2^ ), peripheral vascular disease, and coagulation disorders were excluded from the study.

Computer-generated randomization by the block randomization technique was done by an investigator not involved in patient care to allocate the participants to two groups. Random block sizes were used to reduce the selection bias. Group allocation was concealed using sequentially numbered opaque sealed envelopes before starting the study. The opaque envelope of the recruited patient was opened by the anesthesiologist in the operation theater just before the procedure was done. The arterial cannulation in both groups was performed by the same experienced anesthesiologist, who was well-versed in ultrasound and both cannulation methods with a 20G arterial switch cannula.

Sample size estimation

The determination of the sample size relied on the findings reported by Nam K et al. [10], where they found the first attempt success rate as 94% and 68.2% using the DNTP and LAIP techniques, respectively. With a 95% confidence interval and 90% power, the estimated sample size was 48 for each of the two groups.

Upon the patient's arrival in the operating room, standard monitoring, such as pulse oximetry, non-invasive blood pressure, and electrocardiography, was initiated. The operator had full discretion in selecting the radial artery for cannulation. After anesthesia was induced, the patient's arm was held slightly abducted from the body and a support roll was placed under the wrist to allow dorsiflexion of 30 to 45 degrees. To ensure stability, the wrist was secured to the arm board using tape. After endotracheal intubation, radial artery cannulation was performed under aseptic conditions using either the ultrasound-guided DNTP or LAIP technique.

**Figure 1:**
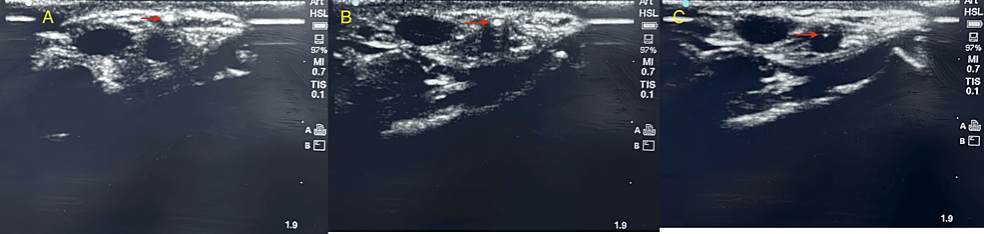
Radial artery cannulation using the DNTP technique The figure shows the advancement of the cannula into the radial artery. A: Hyperechoic needle tip (arrow) is under the skin near the radial artery, B: Needle tip at the wall of the artery, C: The needle tip is advanced to the center of the radial artery. DNTP: dynamic needle-tip positioning

For the DNTP technique, the radial artery was identified using the short-axis out-of-plane approach. An arterial cannula was inserted at a 30° to 45° angle in the center of the probe until the ultrasound image displayed the needle tip as a hyperechoic dot. After that, the ultrasound probe was moved proximally along the arm until the ultrasound image no longer showed the needle tip. Keeping the probe in place, the cannula was moved further toward the radial artery until the needle tip was visible on the ultrasound image once again. This step-by-step procedure was repeated until the tip of the needle was visible in the middle of the lumen of the radial artery. Then, the needle was removed and the catheter was advanced inside the arterial lumen, the transducer tubing was attached, and the arterial pressure waveform was recorded (Figure 1).

For patients in the LAIP group, a long-axis view of the radial artery was obtained and the probe was adjusted to maximize the arterial diameter in the ultrasound image. The needle was inserted where the probe's center line made contact with the skin. During the procedure, the needle was adjusted into the ultrasound plane, and the artery was cannulated utilizing a real-time in-plane technique. 

The cannulation time was defined as the time from the initial skin puncture to the appearance of the arterial waveform on the monitor. A successful first pass was considered as receiving an arterial waveform after only one needle pass through the skin. After threading the catheter through the needle, the transducer tubing was attached, and the arterial pressure waveform was recorded.

Statistical analysis 

The data were recorded in a Microsoft Excel sheet (Microsoft Corporation, Redmond, WA, USA), and statistical software, namely, SPSS 22.0 (IBM Corp., Armonk, NY, USA) was used for the analysis of the data. The continuous variables were expressed as mean ± standard deviation and median (25th, 75th percentiles) while the categorical variables were expressed as counts (percentages). The Kolmogorov-Smirnov test was used to examine the distribution's normality. The ordinal variables and non-normally distributed continuous variables were compared between the groups using the Wilcoxon Mann Whitney-U test while the normally distributed continuous variables were compared using the unpaired student's t-test. Fisher's exact test or chi-square test was used to examine the relationship between categorical variables. A p-value of less than 0.05 was considered significant.

## Results

**Figure 2:**
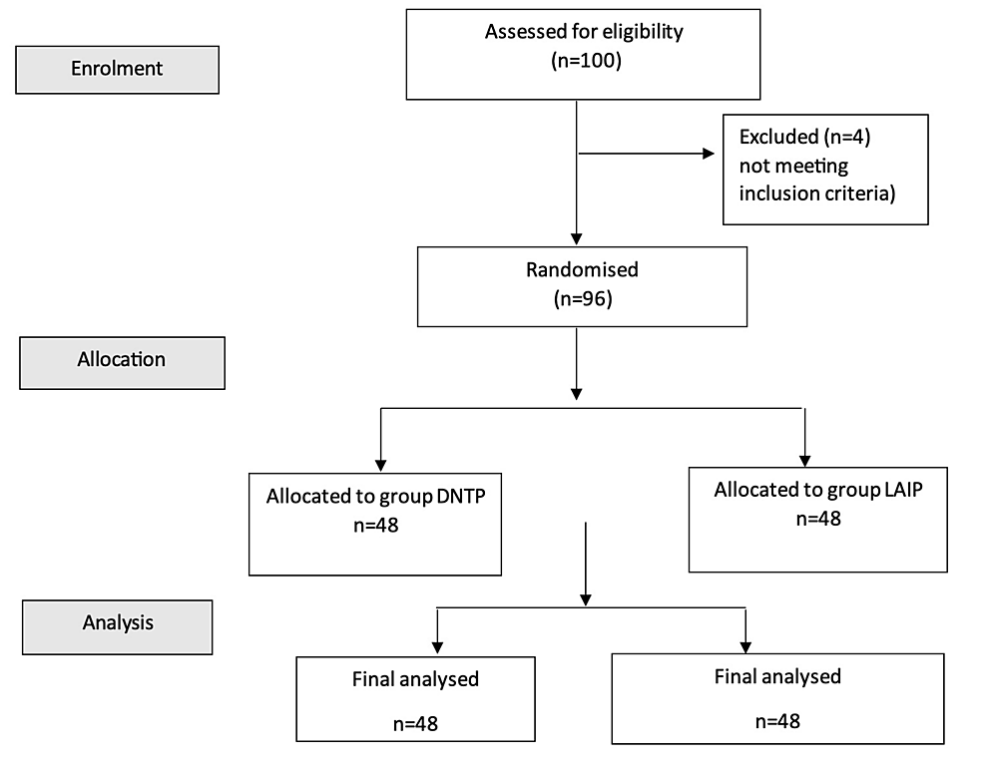
CONSORT diagram showing patient allocation, randomization, and analysis

A total of 96 patients were included in the study (Figure 2).

The demographic data were comparable in both groups (Table 1).

**Table 1:** Demographic data of patients enrolled * chi-square test, # student's t-test, BMI (body mass index), MAP (mean arterial pressure)

Parameters	LAIP (n = 48)	DNTP (n = 48)	P value
Age (Yrs)	41.27±14.74	45.9±13.17	0.603^#^
Sex (Male/Female)	27/21	30/18	0.267*
BMI (kg/m^2^)	24.17±1.54	24.28±1.63	0.367^#^
Baseline MAP (mmHg)	87.44±14.67	88.52±10.03	0.6747^#^

The first-pass success rate was statistically significantly greater in the DNTP group as compared with the LAIP group (97.9% vs 83.3%; p = 0.014) (Table 2).

**Table 2:** First-pass success, number of skin punctures, and time to achieve successful cannulation (seconds) * chi-square test, # student's t-test, $ Fisher's exact test

Parameters	LAIP (n = 48)	DNTP (n = 48)	P value
First-pass success rate	40 (83.3%)	47 (97.9%)	0.014*
Number of skin punctures			0.014*
1	40 (83.3%)	47 (97.9%)	
2	08 (16.7%)	01 (02.1%)	
Time to achieve successful cannulation (in seconds)	26.16±20.22	9.29±3.79	0.001^#^
Overall success within 5 minutes	48 (100%)	48 (100%)	1.000^$^

The number of patients in whom more than one skin puncture was required was greater in the LAIP group compared with the DNTP group (p = 0.014) (Table 2). The time for completion of the procedure was significantly longer in the LAIP group as compared to the DNTP group (26.16±22.22 s vs 9.29±3.79 s; p = 0.001) (Table 2). There was no significant difference in the incidence of hematoma (p = 0.279) and arterial spasm (p = 0.154) between the two groups (Table 3).

**Table 3:** Incidence of complications $ Fisher's exact test

Parameters	LAIP (n = 48)	DNTP (n = 48)	P value
Arterial Spasm	03	01	0.154^$^
Hematoma	02	01	0.279^$^
Thrombosis	00	00	1.000^$^

## Discussion

All patients enrolled in the study underwent successful radial artery cannulation. The DNTP technique appeared to be superior for achieving a first-pass success rate with fewer skin punctures compared with the LAIP technique. Moreover, the time required for successful cannulation was also shorter with the DNTP technique.

The American Society of Echocardiography and the Society of Cardiovascular Anaesthesiologists have both recommended to use ultrasound guidance during radial artery cannulation (category a, level 1 evidence) [11]. There are two basic needling approaches using ultrasound, the LAIP approach and the short-axis out-of-plane (SAOOP) approach, both of which have their advantages and disadvantages. A recent meta-analysis comparing the SAOOP approach with the LAIP technique found that there were no significant differences in first-attempt success rate, total success rate, and cannulation time between the two techniques. However, the rates of posterior vessel wall damage and hematoma were lower with the LAIP technique [12]. According to another systematic review comparing the two procedures, LAIP ultrasound guidance was shown to be superior to SAOOP ultrasound guidance for attaining a higher first-pass success rate with fewer redirections and skin punctures [13].

Recently, a modified ultrasound technique has been introduced using the short-axis approach where the needle tip is advanced in a stepwise fashion, i.e. DNTP. It was first shown to result in a greater success rate for peripheral vascular access when compared to the LAIP view in a phantom study (97% vs 81%) [8].

The primary objective of this study was to assess the first-pass success rate of radial arterial cannulation by the DNTP approach and the LAIP approach. The first pass success rate in the present study for the DNTP group was 97.9% as compared to 83.3% in the LAIP group. The midpoint of the radial artery was precisely identified in the DNTP view as compared with the LAIP view and that may have attributed to this clinical benefit. Earlier studies have also compared the DNTP technique with the LAIP technique. In the study by Nam K et al., the DNTP technique had a greater first-attempt success rate of radial artery cannulation compared to the conventional LAIP technique (94% vs 68%) [10].

In our study, the mean cannulation time required for successful cannulation was significantly less in the DNTP group (9.29±3.79 seconds) as compared to the LAIP group (26.16±20.22 seconds). This might be due to the less time required for localization of the midpoint of the radial artery with the DNTP group as compared to the LAIP group.

With the usage of ultrasound, the identification of the radial artery becomes more feasible. Radial artery cannulation requires successful advancement of the catheter after the puncture of the radial artery. By applying the DNTP technique, after a successful puncture of the radial artery, the needle tip can be advanced sequentially under ultrasound guidance by gradually advancing the needle and the ultrasound probe in turn. This helps in confirming the presence of both the needle and the catheter in the vessel lumen before the catheter is advanced.

It is equally important to reduce the rate of complications associated with arterial puncture. Complications like hematoma, thrombosis, and spasms of the radial artery occurred in a few patients in the present study, as the ultrasonic guidance facilitated the visualization and localization of the radial artery and because of the experienced personnel performing the cannulations. Several studies regarding ultrasound-guided radial arterial cannulation have observed the same results in view of complications [12,14]. The study by Takeshita et al. showed that the DNTP technique reduced posterior wall punctures in small children during arterial cannulation [15].

Hematoma at the puncture site and arterial spasm after a failed first attempt can make arterial cannulation more difficult in subsequent attempts. Therefore, a successful first attempt at arterial cannulation can prevent repeated attempts and ensuing tissue damage.

 Limitations

The major limitation of our study was that patients with hypotension and patients with peripheral vascular disease in whom arterial cannulation is challenging were not included. We did not include pediatric and geriatric populations in whom vascular access is difficult. Ours was a single-centered study comprising 96 participants; a multicentric study comprising a larger sample size will yield much better results.

## Conclusions

We conclude that the ultrasound-guided, short-axis DNTP technique for radial artery cannulation as compared with the LAIP technique may significantly improve the first attempt cannulation, number of attempts, and median cannulation time. This might be due to the better visualization of the needle tip with the DNTP technique as compared to the LAIP technique. Complications like hematoma, arterial spasm, and thrombosis were comparable in both techniques.
